# Morphological and molecular characterization of a marine fish trypanosome from South Africa, including its development in a leech vector

**DOI:** 10.1186/1756-3305-7-50

**Published:** 2014-01-24

**Authors:** Polly M Hayes, Scott P Lawton, Nico J Smit, Wendy C Gibson, Angela J Davies

**Affiliations:** 1 Department of Life Sciences, Natural History Museum, London SW7 5BD, UK; 2 Molecular Parasitology Laboratory, School of Life Sciences, Kingston University, Kingston upon Thames, Surrey KT1 2EE, UK; 3 Water Research Group (Ecology), Unit for Environmental Sciences and Management, North-West University, Potchefstroom Campus, Potchefstroom 2520, South Africa; 4 School of Biological Sciences, University of Bristol, Bristol BS8 1UG, UK

**Keywords:** Fishes, Leeches, Trypanosomes, *Trypanosoma nudigobii*, Life cycle, 18S rDNA sequences

## Abstract

**Background:**

Trypanosomes are ubiquitous blood parasites of marine and freshwater fishes, typically transmitted by aquatic leeches. Phylogenetic studies have been dominated by examples derived from freshwater fishes, with few marine representatives. Furthermore, life cycle studies on marine fish trypanosomes have focused on those of the northern hemisphere. In this investigation, we have examined the life cycle and molecular taxonomy of a marine fish trypanosome from South Africa.

**Methods:**

To locate trypanosome stages, leeches were removed from fishes captured on the west and south coasts of South Africa, and fish blood films and leech squashes were Giemsa-stained and screened; leeches were also examined histologically. To determine whether trypanosome stages in fishes and leeches were of the same genotype, DNA was extracted from Giemsa-stained fish blood films and leech squashes, and from fish whole blood. Fragments of the 18S rRNA gene were amplified by PCR using trypanosome-specific primers and sequenced. Resulting sequence data were compared with each other and with published trypanosome 18S rDNA sequences, and used for phylogenetic analysis.

**Results:**

Trypanosomes were detected in blood films from fishes of the families Clinidae, Blenniidae and Gobiidae. The flagellates ranged in size and staining properties within the films and across fish hosts. In squashes and histological sections of adult and juvenile leeches, identified as *Zeylanicobdella arugamensis*, trypanosome developmental stages were predominantly slender epimastigotes. Sequence data showed that trypanosomes derived from fishes were identical, irrespective of whether they were small or large forms; sequences derived largely from leech epimastigotes were also identical to those obtained from fish trypanosomes. Fish and leech trypanosome sequences fell into a marine fish aquatic clade, and aligned most closely with two trypanosome sequences from marine fishes off Norway.

**Conclusions:**

Combined morphological and molecular methods indicate that the trypanosomes examined here represent a single pleomorphic species, rather than the three species described originally. This species is identified as *Trypanosoma nudigobii* Fantham, 1919 with the leech *Z. arugamensis* as its vector, and *T. capigobii* Fantham, 1919 and *T. blenniclini* Fantham, 1930 are regarded as junior synonyms of the species. Phylogenetic analysis establishes its affinity with marine fish trypanosomes off Norway.

## Background

The life cycles of most fish trypanosomes (Euglenozoa; Kinetoplastea) are unknown, but leeches have been identified as the invertebrate hosts and vectors for some from freshwater fishes [1]. Leeches are also the likely vectors for marine fish trypanosomes, especially in the northern hemisphere [2-9], although proof of such transmission is often lacking.

Prior to 2006, knowledge of marine fish trypanosomes in South Africa was limited to three named species, based on their vertebrate stages [10,11], with additional unnamed trypanosomes noted in brackish water fishes of the family Mugilidae from the region [12,13]. Of the marine species, *Trypanosoma nudigobii* Fantham, 1919 and the smaller *Trypanosoma capigobii* Fantham, 1919 were described from the gobiid, *Caffrogobius nudiceps* (Valenciennes, 1837) (syn. *Gobius nudiceps*), at Kalk Bay on the southern coast [10]. Later, *Trypanosoma blenniclini* Fantham, 1930 was recorded from the blenniid, *Parablennius cornutus* (Linnaeus, 1758) (syn. *Blennius cornutus*) and the clinid *Blennophis anguillaris* (Valenciennes, 1836) (syn. *Clinus anguillaris*) [11]. In addition, larger forms of *T. capigobii* were reported in *C. nudiceps* close to Kalk Bay, at St James [11]. However, in 2006, Yeld and Smit [14] described a trypanosome, *Trypanosoma haploblephari* Yeld and Smit, 2006, from marine elasmobranchs in South Africa, although no developmental stages were detected in leeches taken from the captured fishes. Furthermore, Hayes *et al.*[15] reported briefly trypanosomes resembling those of Fantham [10,11], parasitizing several small marine teleosts in South Africa, with their possible development in an intertidal leech.

In addition to fish trypanosome life cycles often proving problematic to resolve, phylogenetic relationships among these flagellates are often uncertain [16-18]. Freshwater species predominate phylogenetic studies of fish trypanosomes, using 18S rDNA sequences, with only a few marine species included, such as *Trypanosoma boissoni* Ranque, 1973 and *Trypanosoma senegalense* Ranque, 1973 from the sea off Senegal, and *Trypanosoma pleuronectidium* Robertson, 1906 and *Trypanosoma murmanense* Nikitin, 1927, from the sea off Norway [16,18-20]. To date, studies of phylogenetic relationships among fish trypanosomes in southern Africa are limited to freshwater species [17].

In the present study, the marine trypanosomes from teleost and leech hosts reported briefly from South Africa by Hayes *et al.*[15] are fully characterized, together with additional trypanosome material from both types of host taken from a new collection site in the same region. Morphological and molecular methods are applied to resolve the life cycle of a single fish trypanosome, with its phylogenetic relationships inferred from partial 18S rDNA sequences. Finally, comparisons are made between this material and the three marine fish trypanosomes recorded by Fantham [10,11]. The work is novel in that it is the first to identify the vertebrate and invertebrate hosts of a marine fish trypanosome using both morphological and molecular methods.

## Methods

### Fishes, leeches and their initial screening

From Fantham’s records [10,11] and those of Hayes *et al.*[15] it appeared that small intertidal fishes of the teleost families Clinidae, Blenniidae and Gobiidae were mostly likely to be parasitized by the trypanosomes under consideration. Therefore, in the current study, trypanosome material relating to these fish families dating from 2003 [15] was re-examined, and newer 2008, 2010 and 2013 samples were studied, with the same focus.

Material was re-assessed from 51 host fishes, including *Clinus agilis* Smith, 1931 and *Clinus cottoides* Valenciennes, 1836 (Clinidae), and *Parablennius cornutus* (Linnaeus, 1758) (Blenniidae), collected from rock pools at Mouille Point (33°89′93 S, 18°40′39 E), near Cape Town, and Koppie Alleen (34°46′87 S, 20°53′86 E), De Hoop Nature Reserve, on the west (Atlantic Ocean) and south (Indian Ocean) coasts of South Africa respectively, in September and October 2003 [15] (Table 1). Further rock pool fishes (n = 41), including *Caffrogobius nudiceps* (Gobiidae), and *Clinus superciliosus* (Linnaeus, 1758), *Clinus taurus* Gilchrist & Thompson, 1908 and *Pavoclinus graminis* (Gilchrist & Thompson, 1908) (Clinidae), were captured at Tsitsikamma National Park (34°02′07 S, 23°87′60 E), also on the south coast (Indian Ocean) of this region, in April 2008, 2010, and 2013 (Table 1) using the methods of Hayes *et al.*[15], and transported live to a field laboratory.

**Table 1:** Study sites with dates, fish species with size details, trypanosome and leech prevalences

**Site and date**	**Fishes**			**Prevalence of trypanosomes in fishes**	**Prevalence of fishes with leeches (total number of leeches; adults or juveniles)**	**Prevalence of trypanosome stages in leeches (leech adults or juveniles)**	**Reference**
	**Species**	**No**	**TL ± SD (range) in cm**				
Mouille Point October 2003	*Clinus agilis*	1	8	1/1 (100%)	0/1	-	[15]
Koppie Alleen October 2003	*Clinus cottoides*	47	6.4 ± 1.8 (2.6-8.9)	10/47 (21%)	9/47 (19%) (9 leeches; 8 adults, 1 juvenile)	8/9 (89%) (7 adults, 1 juvenile)	[15]
	*Parablennius cornutus*	3	7.2, 8.4, 9.1	2/3 (67%)	1/3 (33%) (1 leech; 1 adult)	1/1 (100%) (1 adult)	[15]
Tsitsitkamma April 2008	*Caffrogobius nudiceps*	6	11.0 ± 0.9 (10.1-12.6)	0/6 (0%)	0/6 (0%)	-	This study
	*Clinus superciliosus*	16	14.0 ± 2.8 (6.4-17.9)	11/16 (69%)	4/16 (25%) (7 leeches; 4 adults, 3 juveniles)	3/7 (43%) (2 adults, 1 juvenile)	This study
	*Clinus taurus*	2	20.6, 15.4	1/2 (50%)	1/2 (50%) (1 leech; adult)	1/1 (100%) (1 adult)	This study
	*Pavoclinus graminis*	1	13.1	0/1 (0%)	0/1 (0%)	-	This study
Tsitsitkamma April 2010	*Clinus superciliosus*	5	13.1 ± 2.2 (9.1-14.9)	3/5 (60%)	0/5 (0%)	-	This study
	*Clinus taurus*	1	23.3	0/1 (0%)	0/1 (0%)	-	This study
Tsitsitkamma April 2013	*Clinus superciliosus*	10	10.7 ± 2.18 (7.8-14.2)	0/10 (0%)	0/10 (0%)	-	This study
**TOTAL**		**92**		**28/92 (30%)**	**15/92 (16%) (18 leeches; 14 adults, 4 juveniles)**	**13/18 (11 adults, 2 juveniles)**	

*No* number of fishes, *TL* fish total length and range in parenthesis, *SD* standard deviation. Reference to the previous and current study.

As in the earlier study, fishes were anaesthetised with clove oil [21] and then examined for leeches, *Zeylanicobdella arugamensis* De Silva, 1963 [15]. These annelids (n = 18, including 10 from 2003 and 8 from 2008, see Table 1) were located mainly on the pectoral and pelvic fins of host fishes, and were removed and maintained singly, or in groups, in fresh seawater [15]. Juvenile gnathiid isopods identified as *Gnathia africana* Barnard, 1914 [22], were found on the fishes at Tsitsikamma, but since no trypanosome stages had been found in these crustaceans previously [15], they played no further part in the current study and were used for other research purposes.

Blood films from 51 fishes captured at Mouille Point and at Koppie Alleen in 2003 [15] were re-examined. Further films were prepared from anaesthetized fishes following removal of blood from the heart or caudal vein using a 23 gauge needle with an attached 1 ml syringe, or by snipping a few gill filaments. Blood films were then fixed in absolute methanol and stained with conventional Giemsa’s stain [15]. Whole blood samples, also extracted by needle and syringe, were placed in sterile 2 ml tubes and fixed in 70% molecular grade ethanol (~ one third whole blood to two thirds ethanol), sealed, frozen at -20°C, and processed subsequently for molecular analysis (see below). Giemsa-stained squashes from 15 leeches (11 adults and 4 juveniles) were prepared up to 32 days post feeding (d.p.f.) on fishes, and haematoxylin and eosin-stained histological sections of a further 3 leeches (2 adults and 1 juvenile) were processed at ~1 d.p.f. [15].

Fish blood films, leech squashes and histological sections were all screened with brightfield or differential interference contrast (DIC) facilities on Nikon Eclipse 80i (Nikon, Tokyo, Japan) or Zeiss Axioskop (Carl Zeiss, Germany) photomicroscopes. Images of films, squashes and sections were then captured with a Nikon DS-5 M camera and Nikon NIS 2.10 image analysis system (Nikon), calibrated to a stage micrometer.

### DNA extraction, PCR and sequence analysis

Following screening, and just prior to DNA extraction, Giemsa stained fish blood films and leech squashes, positive for trypanosome stages, were scraped separately using sterile scalpel blades into 1.5 ml microcentrifuge tubes. DNA from these, and from 8 whole blood samples fixed in 70% ethanol, was extracted using a QIAamp DNA mini kit or Qiagen DNeasy blood and tissue kit (QIAGEN Ltd., UK) according to the manufacturers’ instructions. Once extracted, purified DNA was used as a template for PCR (polymerase chain reaction), using trypanosome-specific 18S rRNA gene primers B (5′-CGAACAACTGCCCTATCAGC-3′) and I (5′-GACTACAATGGTCTCTAATC-3′) to generate sequences of approximately 900 bp [23]. PCR conditions were as follows: initial denaturation at 95°C for 5 min, followed by 35 cycles of 95°C for 1 min, 50°C for 1 min, 72°C for 2 min, and a final extension time of 10 min at 72°C. Sequencing reactions were performed either on PCR products directly, or on gel purified products using an Applied Biosystems Big Dye Kit version 1.1 and run on an Applied Biosystems 3730 DNA Analyzer. Resultant sequences were viewed and edited in Bioedit 7.5.0.2 [24] and identified as trypanosomes using the Basic Local Alignment Search Tool (BLAST) (http://www.ncbi.nlm.nih.gov/blast/Blast.cgi).

### Phylogenetic analysis of sequence data

Published trypanosome 18S rDNA sequences representing 27 trypanosome species which parasitize freshwater and marine animals including fishes, amphibians and reptiles, as well as the monotreme mammal, the duck-billed platypus, were retrieved from GenBank (http://www.ncbi.nlm.nih.gov/blast/Blast.cgi) and aligned with the trypanosome sequences generated from fish blood and leeches in this study. Alignments were performed using the MUSCLE sequence alignment tool (http://www.ebi.ac.uk) and visualised in Bioedit where any final minor adjustments were performed by eye. All phylogenetic analyses were undertaken using MEGA5 [25]. Neighbour Joining (NJ) phylogenies were constructed using the Kimura 2 parameter (K2P) for pairwise distance calculations, as well as a Maximum Composite Likelihood model used to verify the tree topology produced by the K2P model. Character based phylogenetic analyses were also performed including the construction of maximum parsimony (MP) phylogenies as well as maximum likelihood (ML) phylogenies. The ML analysis was performed under the conditions of the K2P model with a four category gamma (G) distribution, as determined using the model test function also in MEGA version 5 [25]. Bootstrap analysis was undertaken with 500 replicates and only those values >50 were shown. In all of the analyses *Trypanosoma lewisi* [GenBank:AJ223566], *Trypanosoma grayi* [GenBank:AJ223565] and *Trypanosoma avium* [GenBank:U39578] sequences formed the outgroup.

This study received the relevant ethical approval (North-West University ethics approval no: NWU-00095-12-A4).

## Results

### General observations on blood parasites in fishes and leeches

The identity, number and length of fishes with trypanosomes (n = 51), collected at Mouille Point and Koppie Alleen in October 2003, and the prevalence of these flagellates in the fishes, as well as leeches, are detailed in Hayes *et al.*[15], but summarized in Table 1. Of the three fish species parasitized, 1/1 *Clinus agilis* from Mouille Point, and 10/47 *Clinus cottoides* and 2/3 *Parablennius cornutus* from Koppie Alleen had trypanosomes, as well as 9/10 specimens of the leech *Zeylanicobdella arugamensis* taken from *C. cottoides* and *P. cornutus* at Koppie Alleen.

A further 41 fishes belonging to four species in three genera were collected from Tsitsikamma in April of 2008, 2010 and 2013 (see Table 1). Of these, *Caffrogobius nudiceps* and *Pavoclinus graminis* were not found to be parasitized by trypanosomes or leeches, but a total of 14/26 *Clinus superciliosus* and 1/3 *Clinus taurus* contained the flagellates, as well as 3/7 leeches from *C. supercilosus* and one leech from *C. taurus* (see Table 1)*.* Blood films from fishes and leech squashes at Tsitsikamma, as previously at Mouille Point and Koppie Alleen [15,26], also contained other haematozoans, but these will be reported elsewhere (Davies *et al.* in preparation).

### Description of trypanosome stages in fishes

**Figure 1:**
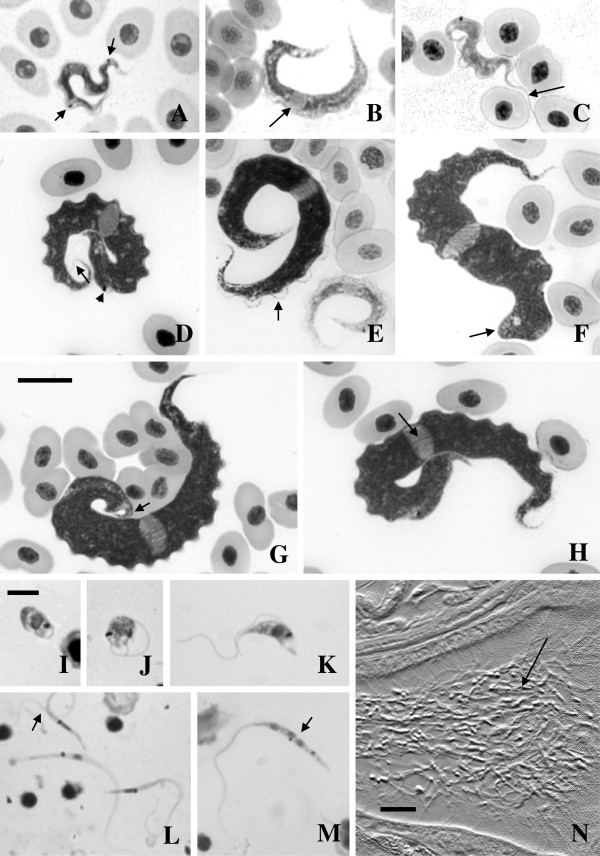
**Brightfield images of Giemsa stained fish blood films (A-H) and leech squashes (I-M); differential interference contrast image of haematoxylin and eosin stained histological section through a leech (N). A**: Small, likely dividing trypanosome with two kinetoplasts (arrows) from *Clinus agilis* at Mouille Point. **B**: Small trypanosome showing the nucleus (arrow) from *Clinus cottoides* at Koppie Alleen. **C**: Small trypanosome demonstrating the flagellum from *Parablennius cornutus* at Koppie Alleen. **D**: Large trypanosome with faintly stained flagellum (arrow) and showing the position of the kinetoplast (arrowhead), from *Clinus superciliosus* at Tsitsikamma. **E**: Large trypanosome (left) demonstrating the undulating membrane (arrow) and small form (right) from *C. superciliosus* at Tsitsikamma. **F**: Large form with bluntly rounded posterior (arrow) from *C. superciliosus* at Tsitsikamma. **G**: Large form with hooked posterior (arrow) from *C. superciliosus* at Tsitsikamma. **H**: Large form demonstrating striae (arrow) from *Clinus superciliosus* at Tsitsikamma. **I**: Amastigote, **(J)** sphaeromastigote, **(K)** short, thick epimastigote, **(L)** slender epimastigotes, one with two flagella (arrow), **(M)** slender epimastigote (arrow) with three nuclei and two kinetoplasts, all from *Zeylanicobdella arugamensis* from Koppie Alleen. **N**: Numerous long slender epimastigotes (arrow) in the dorsal sinus of an adult *Z. arugamensis* from Koppie Alleen. Scale bars: A-H = 10 μm; I-M = 5 μm; N = 20 μm.

These descriptions are taken from re-assessed Mouille Point and Koppie Alleen (2003) material, as well as from blood films prepared in 2008 and 2010 at Tsitsikamma. No trypanosomes were found in 2013 samples from Tsitsikamma. In each trypanosome positive blood film ~5 of these flagellates were normally present, and they varied in size and staining properties. As Fantham [10,11] described, we observed “small” and “large” trypanosomes (blood trypomastigotes) in fish blood films (Figure 1A-H) and, because our largest trypanosomes approached 100 μm in total body length (excluding the free flagellum), we have defined, for ease of comparison with Fantham’s trypanosomes, a total body length of 49.9 μm, or less, as a “small trypanosome” and of 50 μm, or more, as a “large trypanosome”. The morphometric data [27] for these “small” and “large” trypanosome types are shown in Table 2.

**Table 2:** Morphometrics of large (*) and small (^**‡**^**) fish trypanosomes collected at the study sites**

**Fish hosts by species**	**Number measured and morphometrics of fish trypanosomes**								
	**No**	**MA**	**MP**	**MK**	**PK**	**NL**	**BW**	**TBL**	**NI**
Clinus agilis*	7	37.1 ± 2.1	33.0 ± 1.9	21.5 ± 5.0	14.3 ± 4.0	4.0 ± 0.4	9.1 ± 1.4	70.1 ± 2.4	0.9 ± 0.9
		(33.7 - 40.6)	(31.0 - 36.8)	(14.6 - 29.4)	(9.2 - 20.8)	(3.3 - 4.4)	(7.1 - 11.1)	(67.2 - 74.7)	
Clinus. cottoides*	14	36.1 ± 3.5	35.4 ± 4.7	19.7 ± 2.6	18.2 ± 2.8	4.4 ± 0.6	10.4 ± 3.4	71.5 ± 7.2	1.0 ± 1.3
		(31.7 - 42.0)	(24.4 - 42.9)	(14.9 - 24.1)	(13.7 - 22.6)	(3.4 - 5.3)	(6.9 - 18.1)	(59.8 – 81.1)	
*C. cottoides*^‡^	12	15.0 ± 3.9	19.2 ± 2.8	11.8 ± 3.2	7.7 ± 1.9	3.1 ± 0.4	4.5 ± 1.6	34.2 ± 5.8	1.3 ± 0.7
		(9.8 - 22.1)	(15.4 - 25.2)	(3.3 - 16.3)	(4.2 - 10.3)	(2.7 - 3.6)	(2.6 - 7.5)	(25.2 - 46.3)	
Clinus taurus*	1	33.2	29.1	-	-	2.6	6.2	62.3	0.9
Clinus superciliosus*	22	34.6 ± 5.7	35.5 ± 3.6	24.7 ± 4.2	14.0 ± 3.3	3.5 ± 0.8	5.8 ± 1.3	73.3 ± 11.0	1.0 ± 0.1
		(27.0 – 49.2)	(28.1 – 43.2)	(18.9 – 30.3)	(9.3 – 19.2)	(2.2 – 4.8)	(4.1 – 8.5)	(55.1 – 97.7)	
*C. superciliosus*^‡^	15	21.6 ± 1.8	20.8 ± 2.5	12.3 ±1.9	7.9 ±1.2	2.9 ± 0.6	3.6 ± 0.8	42.4 ± 4.1	1.0 ± 0.1
		(18.2 – 24.2)	(15.9 – 24.8)	(10.2 – 13.9)	(6.6 – 8.9)	(1.9 – 4.5)	(2.6 - 5.7)	(35.1 – 48.8)	
*Parablennius cornutus*^‡^	3	12.8, 15.7, 16.5	15.7, 16.1, 19.5	13.5, 13.5, 14.4	4.2, 4.7, 5.1	2.0, 2.5, 2.8	1.4, 2.2, 2.5	31.5, 32.3, 32.5	1.2, 1.0, 1.2

Trypanosome measurements in μm ± standard deviation; range in parenthesis. *Abbreviations*: *No* number of trypanosomes measured, *MA* midnucleus to anterior, *MP* midnucleus to posterior, *MK* midnucleus to kinetoplast, *PK* posterior to kinetoplast, *NL* nuclear length, *BW* body width, *TBL* total body length, *NI* nuclear index (MP/MA). Free flagellum not visible in most individuals and its measurements are omitted; kinetoplast of one large trypanosome from *Clinus taurus* also ill-defined and its morphometrics are also omitted.

*fish with large trypanosomes, ^‡^fish with small trypanosomes.

Large trypanosomes dominated infections in *C. agilis* from Mouille Point, with one small form with two kinetoplasts suspected to be a division stage (Figure 1A); a similar, single, dividing form was also seen in *P. cornutus* at Koppie Alleen. Small and large trypanosomes formed mixed populations in most smears examined from *C. cottoides* at Koppie Alleen (Figure 1B). One of two *P. cornutus* from the same location had mainly small trypanosomes (Figure 1C) and the larger forms were obscured by lying in thick areas of the blood films, and have been omitted from Table 2. At Tsitsikamma, *C. superciliosus* appeared to contain in roughly equal proportions, large, small, or a mixture of small and large trypanosomes (Figure 1D-H). *Clinus taurus* at the same location, however, appeared to be parasitized largely by small trypanosomes, although these were also obscured by lying in thick areas of the blood films, making their measurement difficult (the small forms are also omitted from Table 2).

Small trypanosomes stained pale blue with Giemsa, whereas the larger forms tended to be deeply blue stained, sometimes displaying distinct longitudinal striae, up to 8 or more in number (Figure 1A-C, H). Cytoplasm in both small and large trypanosomes was granulated, often coarsely, while nuclei in both types were normally oval or rectangular, stained pale pink and were wider than long, extending across the full body width (Figure 1A-H). However, in some small trypanosomes, the nucleus was rounded or longer than wide, with its long axis extending along the body length. The nuclear indices (NI values) for small and large trypanosomes were between 0.9 and 1.3 indicating that the nucleus was generally centrally placed or lay just forward of the mid-point of the body (Table 2).

The anterior and posterior extremities of the small trypanosomes were attenuated, often reflexed, or curled (Figure 1A-C). In larger types the anterior end was also attenuated while the posterior extremity was pointed, hooked, bluntly pointed or obtuse (Figure 1D-G), sometimes with coarse granules. The kinetoplast was generally small, rounded, deeply stained and located some distance from the posterior end (Figure 1A, C, D); this distance (PK value) in small forms was approximately half that observed in the larger trypanosomes (see Table 2). In some larger trypanosomes the kinetoplast was surrounded by an unstained halo of cytoplasm.

The undulating membrane was generally well developed in both small and large forms (Figure 1A-H), with eight or fewer undulations in small types and typically eight to 15 waves lying close to the body in larger trypanosomes. Free flagella were generally not easily stained, particularly in the larger forms, with some trypanosomes appearing to have no free flagellum (Figure 1A, B, E-H). When observed, free flagella varied in length, but were typically ~7-9 μm long (Figure 1C, D).

### Description of trypanosome stages in leeches

Hayes *et al.*[15] reported briefly on the developmental stages of trypanosomes in squash preparations and histological sections of the leech, *Z. arugamensis,* from Koppie Alleen. Squashes of 4 adult leeches at 1, 31 and 32 d.p.f., from *C. cottoides*, and of one adult leech at 30 d.p.f. from *P. cornutus*, all contained trypanosomes. Trypanosome stages were also found in histological sections of 2/2 adult leeches (1 d.p.f.) and a juvenile leech (<1 d.p.f.). However, no further details were provided by Hayes *et al.*[15] and thus the trypanosome developmental stages detected in leeches at that time, and subsequently at Tsitsikamma, are described in detail below.

In leech squashes, the developmental stages of trypanosomes included amastigotes, sphaeromastigotes, short thick epimastigotes, longer thick epimastigotes, long slender epimastigotes, a few promastigotes and some metacyclic types. A few large trypomastigote-like forms and a variety of transitional stages were also observed.

Amastigotes were oval or rounded, 5.9 ± 0.6 × 4.0 ± 0.6 μm (range = 5.0-7.0 × 3.3-5.5 μm) (n = 10) and were particularly numerous at 1 d.p.f. (Figure 1I). Division of these stages was not observed. Sphaeromastigotes were also oval or rounded, 5.4 ± 0.9 × 3.7 ± 0.7 μm (range = 3.6-6.7 × 3.1-5.9 μm), with a free flagellum of 10.7 ± 3.8 μm (range = 5.9-19.4 μm) (n = 10). Like amastigotes, they were also most numerous in squashes at 1 d.p.f. and were not seen to divide (Figure 1J).

Short thick epimastigotes (Figure 1K), detected at 1 d.p.f., were 10.5 ± 1.2 × 2.3 ± 0.3 μm (range = 8.4-12.4 × 1.8-2.9 μm) with a free flagellum of 14.9 ± 3.2 μm (range = 8.5-22.5 μm) (n = 25). Their cytoplasm stained pinkish-blue, with the posterior region generally more lightly stained than the anterior. The kinetoplast lay posterior to the nucleus, laterally to this structure, or close to its anterior margin. A short undulating membrane was detected in most, but not all, short epimastigotes. Some were binucleate, while others had two kinetoplasts, suggesting division. Longer, thick epimastigotes, 18.5 ± 4.1 × 2.4 ± 0.4 μm (range = 12.7-23.7 × 2.1-3.2 μm), with a free flagellum of 22.9 ± 5.3 μm (range 10.7-30.3 μm) (n = 10) were found in low numbers in smears at 1, 30–32 d.p.f. Cytoplasm and nuclei were stained as in shorter epimastigotes, with some of these stages also having 2 kinetoplasts.

Long slender epimastigotes, 17.4 ± 3.1 × 1.4 ± 0.2 μm (range = 12.0-26.1 × 0.9-1.8 μm), with a free flagellum of 24.1 ± 4.1 μm (range = 13.2- 31.1 μm) (n = 25), were detected in large numbers, especially at 1 d.p.f. (Figure 1L, M). Their posterior end was often strongly tapered, but blunt posteriors were also present. As in short thick types, the kinetoplast lay just anterior, posterior, or lateral to the nucleus. Binucleate, or sometimes trinucleate, long slender epimastigotes, often with two kinetoplasts and flagella, indicated division (Figure 1L, M). Promastigote stages, with the kinetoplast well forward of the anterior margin of the nucleus, were observed only rarely (at 1 d.p.f.). Early metacyclic trypomastigotes, with the kinetoplast lying posterior, were not numerous either, but were observed at 1, 30–32 d.p.f..

A few large, broad, poorly preserved transitional forms resembling blood trypomastigotes from fishes were seen in leeches squashed at 30–32 d.p.f. (not illustrated). These varied greatly in appearance, and were larger than any other stages observed in leeches. The cytoplasm of these broad forms stained dark blue, with darkly stained granules; the nucleus was lightly stained pink, usually oval or rounded, with the kinetoplast in close proximity. An undulating membrane and free flagellum were visible in some individuals, while others appeared to be undergoing transformation into rounded forms.

Developmental stages of trypanosomes were also found in histological sections of two adult *Z. arugamensis* at 1 d.p.f. and one juvenile at <1 d.p.f. at Koppie Alleen. These included a few amastigotes and short thick epimastigotes, but long slender epimastigotes predominated. Amastigotes were noted in the crop of one adult leech, and these, as well as short thick, and long slender epimastigotes occurred in the intestine, although no attachment to the epithelial lining of this structure was evident. Binucleate, long slender epimastigotes, presumably undergoing division, were seen in the intestine of the second adult leech. Epimastigotes were also observed in the intestine of the juvenile leech, but were less abundant and lacked evidence of division. Numerous long, slender, epimastigotes were located in the dorsal sinus (dorsal coelomic cavity) of both adult leeches (Figure 1N), although none was seen adjacent to the proboscis.

### Sequence identification, alignment and phylogenetic analysis

The longest sequences were obtained from *C. superciliosus* whole blood containing large or small trypanosomes, from Tsitsikamma, and from Giemsa-stained squashes of the leech, *Z. arugamensis,* containing numerous epimastigotes, taken from *C. cottoides* at Koppie Alleen and *C. superciliosus* at Tsitsikamma. The 900 bp 18S rDNA sequences from the fishes or the leeches were identical and general BLAST searches via GenBank showed them all to be most similar to two marine trypanosomes, *T. murmanense* [GenBank:DQ016616] and *T. pleuronectidium* [GenBank:DQ016618] [18].

**Figure 2:**
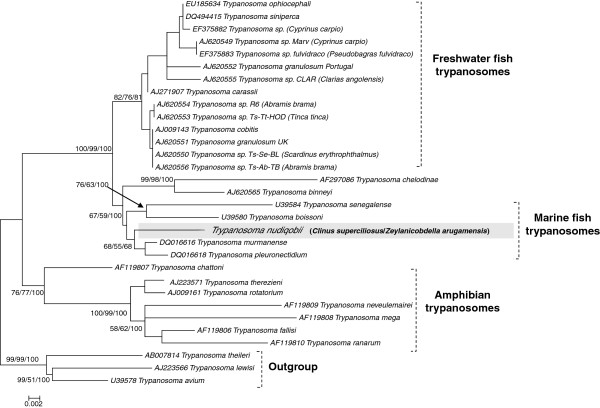
**Phylogenetic position of *Trypanosoma nudigobii *inferred from partial 18S rRNA gene sequences.** Tree topology was identical across Neighbour Joining (NJ), Maximum Likelihood (ML), and Maximum Parsimony (MP) analyses (all with 1000 bootstrap replicates). Therefore, the nodal bootstrap support values for each analyses (NJ/ML/MP) are represented on the ML tree constructed using the K2P model with a four category gamma (G) distribution, showing values of >50%. *Trypanosoma nudigobii* falls within a discrete marine fish trypanosome clade, clustering predominantly with *Trypanosoma murmanense* and *Trypanosoma pleuronectidium*.

As the trypanosome 18S rDNA sequences obtained from *C. superciliosus* and *Z. arugamensis* were identical, only a single sequence [GenBank:KF871790] was used to represent the parasite during phylogenetic reconstruction (Figure 2, where it is identified as that of *Trypanosoma nudigobii* – see the Discussion below). The overall topology of the phylogenetic trees generated was identical irrespective of the analyses employed (Figure 2). All fish trypanosomes comprised a single clade, with amphibian trypanosomes as a sister group. The fish trypanosome clade was further divided into three subclades. Two distinct clades represented the trypanosomes of freshwater and marine fishes, while the third clade contained just two species, *Trypanosoma chelodinae* [GenBank:AF297086] and *Trypanosoma binneyi* [GenBank:AJ620565]*,* which parasitize a turtle and the platypus respectively. *Trypanosoma nudigobii* nested within the marine fish clade and appeared to be a sister species/lineage basal to a small clade which contained *T. murmanense* and *T. pleuronectidium*, confirming a close relationship with these species. The South African trypanosomes thus appeared more closely related to *T. murmanense* and *T. pleuronectidium* than to the Senegalese species *Trypanosoma senegalense* [GenBank:U39584] (previously known as *Trypanosoma trigla senegalensis* [see [18,28]]) and *Trypanosoma boissoni* [GenBank:U39580].

Voucher specimens of trypanosome stages in a blood film from *C. superciliosus* (NMP P 361) collected on 2008/03/30 from Tsitsikamma National Park and a leech squash (NMP P 362) collected on 2003/10/09 from De Hoop Nature Reserve have been deposited in the National Museum in Bloemfontein, South Africa. DNA samples from the leech, *Z.arugamensis,* are to be sequenced, as part of another project, and the resultant sequence data will be made available on GenBank.

## Discussion

Fantham [10] described two trypanosome species from the blood and internal organs of *Caffrogobius nudiceps* in the South African region of Kalk Bay. The larger species, *Trypanosoma nudigobii*, with a short free flagellum and a body with tapering ends, measured 60-85 μm long by 6.6-7.5 μm wide. The undulating membrane was usually narrow, cytoplasm was granular, and normally ten striae (myonemes) were present. An oval, pale staining, nucleus extended across the body and the kinetoplast was small and rounded, often with a pale halo. The smaller trypanosome species, *Trypanosoma capigobii*, also had pointed extremities, but a shorter and narrower body, measuring 42–60 μm long by 2–4.4 μm wide. Five striae were usually observed, and the centrally placed nucleus lay with its long axis parallel to that of the trypanosome body. The undulating membrane arose close to the kinetoplast, which was small and distinct. Later, Fantham [11] found *T. capigobii* in the same host fish (*G. nudiceps*) at St. James, except that the body was 60–80 μm long by 2.7-4 μm wide, and there was “hardly any” free flagellum.

Fantham [11] also observed a trypanosome in *Blennophis anguillaris* and *Parablennius cornutus.* The body of this species, *Trypanosoma blenniclini*, was “long and sinuous with a narrow undulating membrane” and it measured 50*–*77 μm long by 3–7 μm wide. No free flagellum was detected, but numerous striae and coarse granules were recorded in the cytoplasm, rendering it dense in appearance. The nucleus was oval or rounded, and extended across the body width, while the kinetoplast was situated 6–9 μm from the posterior end, which was often bluntly rounded. This third trypanosome species (*T. blenniclini*) was thus intermediate in size between the original descriptions of *T. nudigobii* and *T. capigobii*, and its cytoplasm and nucleus had features of both previously named trypanosome species.

In the current case, trypanosomes were located in a clinid (*Clinus agilis*) captured at Mouile Point, which lies west of Kalk Bay and St James, and in further clinids (*Clinus cottoides*, *Clinus superciliosus* and *Clinus taurus*) and a blenniid (*P. cornutus*) at Koppie Alleen and/or Tsitsikamma, which both lie east of Fantham’s collection sites [10,11]. Surprisingly, however, no trypanosomes were located in *C. nudiceps*, the type host of *T. nudigobii* and *T capigobii*, although present in *P. cornutus*, one of the type hosts of *T. blenniclini*[10,11]*.* In addition, it was evident that trypanosomes were detected in fishes in October 2003 and April 2008 and 2010, but not in April 2013. This lack of trypanosomes in April 2013 reflected earlier observations in South Africa, when no flagellates were found in intertidal fishes along the southern coast between 1999 and 2003 [29-33].

Although “small” and “large” trypanosomes had been separated by total body length (TBL) in the current study (Table 2), there was clearly overlap among the TBL of small or large trypanosomes across the five species of fish hosts parasitized (Table 2). There was also overlap between the highest values in smaller forms and the lowest values in large forms for midnucleus to posterior, midnucleus to kinetoplast, nuclear length, and body width (MP, MK, NL and BW values) [27] (see Table 2). Some trypanosomes resembled Fantham’s smaller *T. capigobii*[10] in size, having a mean body length up to 42.4 μm and body width up to 4.5 μm. Others more closely resembled *T. nudigobii*, the larger *T. capigobii* and *T. blenniclini*[10,11], having a mean body length of 73.3 μm, although mean body width (up to 10.4 μm in *C. cottoides* trypanosomes) could exceed that of Fantham’s three species*.* The body shape of the trypanosomes matched those of Fantham’s three species, the free flagellum also tended to be short or not clearly visible, cytoplasm was granular and striae were often prominent, the nucleus was rounded to oval, and the kinetoplast small and rounded, and sometimes surrounded by a clear halo. Commonality was thus observed among the morphometric features of the current trypanosomes and all three of Fantham’s [10,11] species, suggesting that they might all belong to one pleomorphic species, with the smaller trypanosomes representing immature stages of the larger forms. The suspected division stages amongst the smaller trypanosomes observed in two fish species appeared different from the traditional patterns of division seen in other fish trypanosomes [1,34], but further observations are required before any conclusions can be drawn.

There were no likely vectors of South African marine fish trypanosomes reported by Fantham [10,11], but Hayes *et al.*[15] briefly noted trypanosome stages in the leech *Zeylanicobdella arugamensis*. Since most leeches were taken from infected fishes, the developmental forms of trypanosomes found in leech squashes and histological preparations were likely those of flagellates detected in fish blood films. These stages were found in both a juvenile and adult leeches, suggesting that trypanosomes were acquired at a young age and retained into adulthood in these annelids.

The developmental forms in *Z. arugamensis* were typical of those reported from marine leeches in the northern hemisphere [2-7,35,36]. Their variety and their distribution days post feeding (d.p.f.) suggested a mixture of old and new infections. Dividing epimastigotes were evident at 1 (old infection?), 30, 31 and 32 d.p.f (recent infections?), suggesting that these are the dominant divisional stages of the South African trypanosomes. Epimastigotes were also the main divisional stages of *Trypanosoma rajae* Laveran and Mesnil, 1902 in *Pontobdella muricata* (L.) [35,36], in contrast to the amastigotes and sphaeromastigotes of *Trypanosoma giganteum* Neumann, 1909 in *P. muricata*[4], *Trypanosoma murmanensis* in *Johanssonia* sp*.*[5] and *Trypanosoma cotti* Brumpt and Lebailly, 1904 in *Calliobdella punctata* van Beneden and Hesse, 1863 [6]. The tetranucleate stages reported by Robertson [35] were not observed.

In histological sections of the leeches examined in the present study, amastigotes occurred in the crop, and both these and a variety of epimastigote stages were observed in the intestine. The few metacyclic trypomastigotes seen in squashes were not identified in sections. Robertson [35,36] and Khan [5,7] described a range of polymorphic forms from the intestine of leeches in what they referred to as the middle stages of digestion. Amastigotes and sphaeromastigotes were reported from the crop during the early phase of digestion, while metacyclic trypomastigotes were generally observed in the proboscis following complete digestion of the blood meal [5,7,35,36]. Brumpt [3] also reported a variety of stages of *T. cotti* from the intestine of leeches, but, as in our study, none was observed in the proboscis. As reported by Hayes *et al.*[15], migration to and invasion of the leech proboscis sheath by fish trypanosomes has not been elucidated fully. However, large numbers of long slender epimastigotes were observed in the dorsal sinus of *Z. arugamensis*, a rhyncobdellid leech [37]. This sinus, which includes the dorsal blood vessel, and connects through a series of sinuses and ducts to the medial haemocoelomic sinus adjacent to the proboscis, may thus be the means by which the flagellates are transported, or migrate to the proboscis sheath [15].

Analysis of the 18S rDNA sequences derived from several fish species revealed only one type of sequence was present, irrespective of whether the fishes contained small or large trypanosomes. Identical sequences were secured from Giemsa-stained smears containing predominantly epimastigote stages taken from leeches, *Z. arugamensis,* captured on trypanosome-parasitized fishes at Koppie Alleen and at Tsitsikamma. These results demonstrated that only one pleomorphic trypanosome species was likely present in the fishes and leeches sampled. In addition, since only trypanosome developmental stages, especially epimastigotes, were found in the leech squashes used for DNA analysis, that is, no blood trypomastigotes, the matching sequences indicate that *Z. arugamensis* must be the vector for the fish trypanosome, at least at Koppie Alleen and at Tsitsikamma.

The trypanosome sequences generated in this study fell into a discrete clade consisting of trypanosomes that parasitize marine fishes. However, they appeared more closely related to *Trypanosoma murmanense* and *Trypanosoma pleuronectidium,* both trypanosomes of marine teleosts, especially Atlantic cod, *Gadus morhua* L. off Norway [18], rather than to the two African marine fish trypanosomes in this clade, *Trypanosoma senegalense* and *Trypanosoma boissoni*, from a teleost and an elasmobranch respectively in Senegal [18,28]. The data in the current study support the existence of a predominant fish clade with distinct marine and freshwater subclades, as suggested by Gibson *et al.*[16], Kalsbakk and Nylund [18] and Gu *et al.*[20]. However, an aquatic tetrapod subclade containing *T. binneyi*, from the platypus and *T. chelodinae* from turtles, also falls within the clade of trypanosomes from marine and freshwater fishes, and this occurs in other phylogenetic analyses such as those of Karlsbakk and Nylund [18] and Gu *et al.*[20]. The seemingly anomalous phylogenetic position of this tetrapod clade could simply arise from insufficient taxon sampling and gene sequence information, leading to poor phylogenetic resolution. But more interestingly, it could indicate host swapping leading to parasite species divergence; a lack of fidelity of leech vectors, feeding on a multitude of vertebrate host species, might explain this. Although this separation of the fish clades was not the focus of the current study, only through further sampling with a greater number of species will this issue be resolved.

## Conclusions

Our observations strongly suggest that, despite the variety of trypanosome morphometric types observed by Fantham [10,11] and particularly by us in the current study, the marine fish trypanosomes are likely of one genotype and thus, one pleomorphic species, with the leech *Z. arugamensis* as its vector. We suggest, therefore, that all three species of Fantham [10,11] and our trypanosomes are *Trypanosoma nudigobii*, Fantham’s first named species, and we recommend the following nomenclatural correction:

*Trypanosoma nudigobii* Fantham, 1919 (syn. *Trypanosoma capigobii* Fantham, 1919; syn. *Trypanosoma blenniclini* Fantham, 1930) in the marine fishes *Caffrogobius nudiceps* (type host), *Blennophis anguillaris*, *Clinus agilis, Clinus cottoides*, *Clinus taurus* and *Parablennius cornutus,* and the leech (vector), *Zeylanicobdella arugamensis.* As far as we are aware, this is the first study to link the vertebrate hosts and vector of a marine fish trypanosome by morphological and molecular means.

## Competing interests

The authors declare that they have no competing interests.

## Authors’ contributions

All authors conceived and designed the project, participated in general data analysis and in drafting the manuscript. PMH, NJS and AJD carried out the field work, prepared and examined films, squashes and histological material, and PMH, SPL and WCG conducted most of the molecular analysis. All authors read and approved the final manuscript.
